# Toward an Asbestos Ban in the United States

**DOI:** 10.3390/ijerph14111302

**Published:** 2017-10-26

**Authors:** Richard A. Lemen, Philip J. Landrigan

**Affiliations:** 1 National Institute for Occupational Safety and Health (retired), Washington, DC 20024, USA; richard@ralemen.org; 2 Department of Environmental Health, Rollins School of Public Health of the Emory University, 1518 Clifton Rd., Atlanta, GA 30322, USA; 3 Collegium Ramazzini, Castello di Bentivoglio via Saliceto, Bentivoglio, 340010 Bologna, Italy; 4 Environmental Medicine and Public, Icahn School of Medicine at Mount Sinai, 1 Gustave L. Levy Pl, New York, NY 10029, USA; 5 Division of Surveillance, Hazard Evaluations and Field Studies, National Institute for Occupational Safety and Health, 1090 Tusculum Ave., Cincinnati, OH 45226-1988, USA

**Keywords:** asbestos, regualtion, mesothelioma

## Abstract

Many developed countries have banned the use of asbestos, but not the United States. There have, however, been multiple efforts in the US to establish strict exposure standards, to limit asbestos use, and to seek compensation through the courts for asbestos-injured workers’. In consequence of these efforts, asbestos use has declined dramatically, despite the absence of a legally mandated ban. This manuscript presents a historical review of these efforts.

## 1. Introduction

“*Asbestos dust is, perhaps, the best-documented industrial toxin in history*…”.

[1]

Asbestos kills 12,000–15,000 Americans each year and results in innumerable cases of disease and disability [2]. It is reported that in the United States in 2015, there were 2597 deaths due to mesothelioma, representing about 15–20% of overall asbestos-related deaths [3]. The majority of asbestos-related deaths other than those caused by mesothelioma are due to lung cancer (≈60%) and asbestosis (≈10%), with additional deaths due to cancers of the larynx, ovary and complications of asbestos-related non-malignant respiratory diseases. There is evidence that asbestos exposures are related also to other cancers including stomach, pharynx and colorectal cancer [4]. The overall scientific consensus supports a finding that asbestos is a proven human carcinogen and that there is no safe level of asbestos exposure [4,5].

Many developed countries have banned the use of asbestos, but not the United States. There have, however, been multiple efforts in the US to establish strict exposure standards, to limit asbestos use, and to seek compensation through the courts for asbestos-injured workers’. The National Institute for Occupational Safety and Health (NIOSH) has led these efforts and in 1976 was the first US federal agency to recommend a ban on asbestos in the workplace [6]. In consequence of these initiatives, asbestos use has declined dramatically in the United States, despite the absence of a legally mandated ban.

## 2. Historical Use of Asbestos in the United States

The earliest recorded discovery of asbestos in the United States was on Belvidere Mountain near Lowell, VT before 1823 [7,8]. In 1825, it was reported in Vermont that “*asbestos, both amianthus* (meaning fine, silky asbestos fibers) *and the common variety are abundant, and the fibres are sometimes uncommonly long*” [9]. The Vermont deposits are located in the northwestern part of the state near the southern end of the serpentine mineral belt that runs north from Vermont through Quebec, Canada. Not much interest was shown in these asbestos deposits in the early 1800s, because commercial mining of asbestos did not begin until the 1890s when chrysotile was mined from open pits at three locations on Belvidere Mountain near the towns of Eden and Lowell [7,10].

Importation of asbestos fibers into the United States began to increase in the mid-1800s. According to the 1869 Encyclopedia Americana, Mr. H.W. Johns, founder of the H.W. Johns Manufacturing Company,

“*…made known to the world his discovery of the practical value of this remarkable mineral, and the nature of his patented inventions. That he has labored intelligently in this comparatively new field is proven by a gratifying success and a world-wide reputation, for his asbestos products are in use wherever materials for structural and mechanical purposes are employed.*”

[7,11]

The first patent for an asbestos product was “Improved Compound for Roofing and Other Purposes” [12].

By 1874, the H. W. Johns company developed a pipe covering made from layers of felts, paper, and asbestos, which “*formed one of its most popular and valuable applications*” [11]. When asbestos was discovered on Staten Island, “*it became routine to see in front of the Company’s new headquarters at 87 Maiden Lane a line of farmers’ wagons bringing in asbestos from nearby deposits*” [12]. According to Paul Brodeur, in his excellent *New Yorker* article of 1968, it was:

“*Small wonder, then, that when asbestos, which had been known to the ancients as ‘the magic mineral,’ was in effect, rediscovered less than a hundred years ago in the age of industrial expansion, it was put to work.*”

[13]

Brodeur pointed out that:

“*Asbestos is the only mineral that can be woven into cloth, and its fibrous structure is, if anything, even more amazing than its remarkable ability to withstand heat. In fact, if it were not for the electron microscope, the extent to which asbestos is fibrous would be difficult to believe, for there are approximately a million individual fibrils lying side by side in a linear inch of chrysotile asbestos, whereas only thirty-eight hundred glass fibrils, such as those found in various insulation material, or six hundred and thirty human hairs can be aligned along the same distance. Moreover, in addition to their extreme fineness, high tensile strength, unusual flexibility, spinnability, and resistance to heat and the elements, asbestos fibres possess great powers to adsorb and to filter.*”

[13]

On 13 January 1906, the asbestos company Johns-Manville ran a full-page advertisement in *The Saturday Evening Post* proclaiming that asbestos “*Serves More People in More Ways than any Institution of its kind in the World.*” Products included in this advertisement were designed for the home builder, the industrial and commercial builder, automobilists, and steel companies for their large furnaces. [14]. “*And while the asbestos industry is comparatively undeveloped, it must be rated as one of the great industries of the world*” [15].

By the 1960s and 1970s, over 700,000 tons of asbestos were consumed annually in the U.S. [16]. But because of regulation and selected bans on specific applications resulting from asbestos’ known health effects coupled with vigorous efforts through the courts to seek legal compensation for asbestos-injured workers, U.S. asbestos consumption decreased to 14,600 tons by 2000, slightly lower than that at the turn of the 20th century. By 2000, most U.S. manufactures had ceased production of asbestos-containing products [16]. Surprisingly, however, in 2015 the U.S. was still importing 343 tons of chrysotile asbestos each year—95% of it from Brazil and the remainder from Russia. The chloralkali industry currently accounts for nearly all U.S. asbestos consumption [17]. 

## 3. Health Impact and Its Effect on Regulation and Banning

The health impacts of asbestos have been well documented for over a century [18,19,20]. In 1918 the first descriptions of asbestos disease were published, showing X-ray changes among 15 individuals exposed to asbestos in the *American Journal of Roentgenology* [21]. Shortly thereafter both American and Canadian insurance companies began refusing to insure asbestos workers because of the high costs associated with asbestos workers’ risk of developing disease [22].

During the 1920’s, *asbestosis* was coined as the name for the form of pneumoconiosis (a dust disease affecting the lungs) affecting asbestos-exposed persons and curious bodies, later known as asbestos bodies were first described [23,24] (today it has been shown that asbestos bodies can form in extra-pulmonary sites, such as the liver and spleen [25]. The first official workers compensation claim for asbestos-related disease in the United States was filed in 1927 in Massachusetts for a foreman in the weaving department of an asbestos plant. Also, in that year, the first fatal case of uncomplicated asbestosis was reported to the Medical Society of South Carolina [26]. Throughout the 1920s and 1930s attention to the hazards of asbestos was growing, and on 14 January 1928, the journal of the largest medical organization in the U.S. *The Journal of the American Medical Association* ran an editorial about pulmonary asbestosis reporting the dangers of inhaling asbestos and the resultant disease asbestosis. This editorial cited Cooke’s case from 1927, urging

“*Nevertheless, asbestosis, because of its dangers and its unique pathologic features, deserves more attention than it has had.*”

[27]

In 1930, the first epidemiological study on a cohort of asbestos textile workers was reported in the *Lancet*, a joint American and British medical journal. The findings supported a causal relationship between exposure to asbestos and the lung disease, asbestosis. The study also concluded that asbestosis was a preventable industrial disease [28,29]. The findings of this British study were also published in the Harvard University sponsored *Journal of Industrial Hygiene* [30].

In 1930, the *Journal of the American Medical Association* alerted physicians that asbestosis was linked to working with asbestos and had been introduced into the workmen’s compensation act by the British Parliament [31]. Additionally, the trade journal *Asbestos* made reference to asbestosis, stating that the U.S. Bureau of Labor Statistics urged suppression of asbestos dust as a prime method of its prevention [32]. 

Internationally, the International Labour Office, an organization of which the U.S. was a member, published in its *Encyclopaedia of Hygiene, Pathology and Social Welfare* a discussion on asbestos. This authoritative publication described the dangers of asbestos, recommended industrial hygiene practices, and discussed asbestos related legislation. This report described how U.S. and Canadian insurances companies generally refused asbestos workers insurance coverage due to the high risks of asbestos workers developing disease [33]. The U.S. Bureau of Labor reported:

“*The steadily increasing use of asbestos in industrial processes has created a new occupational risk and added to the list of industrial lung affections a new form of chronic pulmonary fibrosis.*”

In 1931, the *U.S. Bureau of Labor Bulletin* stated how important controlling airborne asbestos dust was in the asbestos industry. The Bulletin recommended the application of localized exhaust ventilation at dust producing points; substitution of enclosed methods for hand conveyance and dusty handwork; the use of wet methods; and prohibiting young persons from working in especially dusty asbestos work areas [34].

A 1935 study of U.S. asbestos textile workers reported that a shocking 87% of workers with over 15 years’ work in the industry had X-ray changes to their lungs [35]. The authors found that airborne asbestos dust could be reduced by as much as 75% through engineering controls. But the authors opined that any further airborne asbestos dust control would be cost-prohibitive and stated that “*it was not practicable as yet to establish standards for the asbestos dust content of air*” [35].

By the mid-1930s it was already “*believed that at any stage of its processing sufficient exposure to asbestos dust can cause a definite pulmonary disability, asbestosis, with characteristic symptoms and chest X-rays*” [29,36]. It was already appreciated by that time that there were substantial differences in susceptibility of disease: “*Thus, the length of exposure that will produce the disease varies with the working conditions and individual susceptibility*” [36]. It was recognized that this variation could be a hindrance to setting one standard that would be protective for all workers.

Cancer in asbestos exposed workers first became a concern in the mid-1930s when reports of lung cancers in workers with asbestosis emerged in both the U.S. and U.K. [37,38].

The widely read 1940 *Encyclopedia Britannica* stated, “*There are two known harmful dusts, silica (Silicon Dioxide) and asbestos*” [39]. By 1942 several states had adopted legal standards meant to regulate airborne dust containing asbestos at a level of 5 mppcf (millions of particles per cubic foot [air]) (California, Colorado, Massachusetts, Michigan, North Carolina, Oklahoma, and Pennsylvania) and 15 mppcf in South Carolina [40].

The advent of World War II brought an acceleration in the use of asbestos for insulating warships and other war-related equipment. In the U.S. Navy asbestos was used for insulating piping, in gaskets and packing in valves, and in insulating high temperature steam turbines. The Navy recognized a need for detecting asbestos-related medical changes in its sailors as well as in shipyard workers. A 1941 survey of pipe shop workers in the New York Naval Yard where respirators, local exhaust systems, and wet down techniques were used detected no asbestos-related disease [41]. Phillip Drinker, a health consultant to the United States Maritime Commission, stated: “*The pressure to turn out ships is great—it should be for the need is urgent—and often we must condone practices that we would not accept in peacetime*” [42]. In a cross-sectional medical surveillance study of four naval shipyards, conducted during World War II by the U.S. Navy and the U.S. Maritime Commission, three cases of asbestosis were found among pipe coverers. All three of these workers with asbestosis were estimated to have been exposed to asbestos-containing dust at levels above the recommended level of 5 mppcf. As a result, the authors concluded pipe covering was a safe occupation when exposures were kept under 5 mppcf [43]. However, because the great majority of the workers in the study had less than 5 years of exposure, the detection of asbestosis of longer latency was unlikely. 

In 1944, in discussing hazardous dusts, including asbestos and the disease asbestosis, the industrial journal, *Heating and Ventilation stated*:

“*Very few data are available on the relation of dust concentration to the incidence of the disease. No minimal safe concentrations have yet been set up and information is scant as to the conditions in those plants where a hazard is known to exist. It has been determined, however, that the asbestos dust encountered in fabricating plants is free of silica when air is floated at the breathing level and that the danger of the dust increases as the quality or length of the fibers increases.*”

[44]

Wilhelm Hueper, a well-recognized toxicologist and first Director of the Environmental Cancer Section of the National Cancer Institute, reported that lung cancer was found in 17–20% of autopsied cases where asbestosis was present, usually occurring 14–20 years from first exposure to asbestos. He recommended “*sanitary measures*” be used to reduce the hazard of asbestos in the workplace and recommended “*workers dying from asbestosis should be subjected to a post–mortem examination including a histological study of the pulmonary lesions, as multiple neoplastic manifestations in asbestosis may be mistaken for tuberculosis*” [45]. In 1944, and again in 1949, the American Medical Association highlighted concern about the risk of asbestos related cancer publishing an editorial saying that asbestos was among the known or suspected occupational cancers [46,47].

In 1952 the *Encyclopedia Britannica* updated its section on asbestos, and noted that:

“*Cancers of the respiratory tract involving the mucosa of the nose, the antral cavities, the larynx, the bronchi and lung occur in workers who are exposed to the inhalation of chrome salts, asbestos dust and nickel carbonyl.*”

[48]

Dr. Hueper continued to expound on the carcinogenic risk of exposure to asbestos saying:

“*The evidence indicating the existence of causal relations between asbestosis and cancer of the lung has received additional support during recent years from several countries (United States, Canada, Great Britain, Germany, and France)*” and observed that when exposure to asbestos is equal between the sexes so is their “*liability to cancer*”

[49]

Two studies by British researchers reported strong epidemiological evidence of the link between lung cancer and asbestos at the location where asbestosis generally occurs. One of the studies mentions pleural cancer [50,51]. The E.I. DuPont De Nemours & Company published a book stating:

“*Pulmonary carcinoma has been observed with such high frequency in employees of the asbestos industry that a causal relationship has been accepted by most authorities.*”

[52]

A major breakthrough in understanding the rate of diseases caused by asbestos occurred in 1960, when a large series of case reports was published involving the rare pleural tumor (mesothelioma) among 33 individuals (22 males, 11 females). All but one of the reported cases had a common exposure to crocidolite asbestos in a mining region of the NW Cape of South Africa. Eighteen were born in the vicinity of the mines and two had arrived in the area as infants. Eleven had exposure to the dust from the vicinity of the mines as children, but several of the cases had no mining experience and were presumed to be exposed due merely to living near the mines [53]. By 1963 Wagner had collected 120 mesothelioma cases which he reported to the 14th International Congress on Occupational Health [54].

Following these case reports from South Africa, the first epidemiological study was conducted in the U.S. showing two peritoneal mesotheliomas where none were expected among workers in a company using mixed forms of asbestos. Additionally, 39 cases of asbestosis were reported, along with 19 cases of lung cancer where only 5.61 were expected. This was consistent with findings from other reports occurring among asbestos exposed workers [55]. A second larger U.S. epidemiological study of 632 insulators exposed to asbestos found 45 died of lung cancer compare to 6.6 expected; 12 of asbestosis; and four of mesothelioma (three pleural, one peritoneal) [56]. These two well conducted epidemiology studies solidified the epidemiological evidence for a strong causal link between mesothelioma and exposure to asbestos. 

During this same time period, the possibility of enforcing a common exposure limit was questioned, because traditional dust suppression techniques used in general manufacturing of asbestos products were not easy to implement for construction and other outdoor work situations. Thus, calls for better prevention techniques were sought [57]. In a conference of the New York Academy of Sciences on the biological effects of asbestos, representatives of the asbestos industry agreed the only way to prevent asbestos-related cancers was to eliminate its use [58]. Epidemiological studies, case reports, and toxicological studies continued to show the strong relationship between asbestos exposure and disease.

## 4. Efforts to Regulate and Ban Asbestos in the United States Prior to Passage of the Occupational Safety and Health Act of 1970 

With the increasing use of asbestos during the first two-thirds of the 20th Century and growing evidence of its detrimental effects to health, the U.S., following the United Kingdom’s 1931 regulations for control of asbestos, decided it was time to do so for its own asbestos workforce [59,60]. As the asbestos manufacturing industry was growing in the 1930’s, a number of steps were necessary to control asbestosis. First, reducing the concentrations of airborne dust, followed by conducting medical surveillance of workers, applying medical controls, and/or applying personal controls (personal control in the 1930s was basically a method to eliminate workers thought to be susceptible to developing dust diseases due to both active or healed respiratory or cardiac conditions such as tuberculosis. In other words, selecting only the most physically fit to work in the industry and through periodic medical examinations maintain only those most fit to continue work in the industry).

The first U.S. proposed guidance limit based on medical effects related to dust measurement came from a study by the U.S. Public Health Service (USPHS) published in 1938 where asbestos dust concentrations were measured and then correlated with health findings. Thus, a level of 5 mppcf as a time-weighted-average was selected as the appropriate guidance concentration even though 3 cases of asbestosis were found in workers having worked below this concentration. Though the authors reported limitations in their study, including the majority of workers had worked less than 5 years and only 12% (*n* = 66) had worked more than 10 years, they concluded: “*It appears from these data that if asbestos dust concentrations in the air breathed are kept below this limit new cases of asbestosis would not appear*” Perhaps because that latency was too short for observation of long term effects like cancers or other long latent diseases, the authors cautioned that the recommended 5 mppcf guidance limit “*may be regarded tentatively as the threshold value for asbestos until better data are available*” [60]. Unfortunately, “*as so often happens, many of the disciples conveniently ignored the limitations expressed by the masters*” [7]. Others warned: “*The idea of adopting standard of permissible dustiness for each harmful dust has a medicolegal appeal that is not at all justified by the data available today*” [36].

This USPHS guidance concentration remained the recommendation for the next 30 plus years, and was adopted by non-governmental organizations, such as the American Conference of Governmental Industrial Hygienists (ACGIH) in 1946 as their Threshold Limit Value (TLV) which did not change until 1968 when they recommended it lowered to 2 mppcf or 12 fibers/milliliter [61]. Dr. Herbert Stokinger of the USPHS, and longtime member of the TLV committee, gave two cautions concerning the TLVs at ACGIH’s 1956 annual meeting in Chicago. Stokinger stated that:

“*the threshold limits are nothing but educated guesses,” and “there is still one group of substances for which some method should be devised for establishing safe air standards—the industrial carcerigens (sp.).*” He recommended “*…adding a safety factor for carcinogenicity. The magnitude of the safety factor is suggested to be from 100 to 500.*”

[62]

Stokinger, who headed the TLV committee from 1962 to 1977 [63] subsequently changed his earlier position and ignored the evidence that asbestos was a carcinogen, including the widely accepted epidemiological study of Doll [50]. These misguided analyses halted efforts in the United States to stop the growing epidemic of asbestos-related disease until after the passage of the Occupational Safety and Health Act of 1970. 

Between 1946 and the passage of the Occupational Safety and Health Act in 1970, U.S. governmental agencies, and multiple states adopted the 5 mppcf recommended guidance including through various legislative Acts or by promulgating regulation. Thus under the 1951 Walsh-Healey Act, many safety provisions in addition to the air concentration level were included such as requiring evaluation of the design and efficiency of control equipment and devices [64]. The Longshoremen’s Act of 1960, covering all longshoremen, adopted the 5 mppcf level [65,66]. In 1967, the Longshoremen’s Act was the first to accept a new counting method, developed by the British to replace the old gravimetric particle measurement technique for fiber counting, of 12 fibers/mL representing approximately 2 mppcf [67]. In this new method only fibers meeting the criteria of being greater than 5 μm in length with an aspect ratio of 3:1 or greater were counted [68]. In 1969, the Walsh-Healey Act did likewise, adopting 12 fibers/mL and 2 mppcf as their asbestos standard [69]. In April 1971, in the same month as the U.S. Occupational Safety and Health Act came into existence, the ACGIH followed suit and adopted a new guidance recommendation for asbestos of 5 fibers/mL [70].

## 5. Impact of the Occupational Safety and Health Act on Regulation and Banning of Asbestos

In the 40 years prior to the passage of the Occupational Safety and Health Act, much information was discovered about the deleterious effects of asbestos. By 1970 it was unquestionably known that asbestos caused asbestosis, lung cancer, mesothelioma; and not only to workers with occupational exposures but also to their families and others who had received para-occupational and environmental exposures. Yet Dr. Irving Selikoff, one of the foremost experts on asbestos-related diseases, testified to Congress in 1970:

“*It is depressing to report, in 1970 that the disease that we knew well 40 years ago is still with us just as if nothing was ever known.*”

[71]

The passage of the Occupational Safety and Health Act of 1970 was probably the most important impetus for catalyzing action to prevent asbestos-related diseases in the U.S. Prior to passage of the OSH Act, safety and health in workplaces had been an uncoordinated hit and miss approach by 50 individual states and multiple U.S. territories. 

Commenting on the effectiveness of asbestos exposure guidance standards up to 1970, S.A. Roach of the University of London stated to the New York Conference on Biological Effects of Asbestos:

“*5 million particles per cu. Ft., are simply standards, although I hope I did not use the word ’safe.’ These are standards which are actually used, although they are not ever expressed as being safe standards.*”

Roach further stated even if the standard were dropped to 2 million particles per cu. ft., this would not be a “*perfectly safe level of dust*” [72]. Notably, a worker would not see *any* dust in the air until reaching a concentration of between 20 to 40 mppcf [73]. 

Warren Cook, founding member of the American Industrial Hygiene Association (AIHA) earlier emphasized: *"This (5 mppcf level) is a very small concentration, so small in fact that the condition may look good even to a critical eye and still present an exposure greater than this low limit*” [74]. Dr. Clark Cooper, Chief of the Division of Occupational Health in the USPHS (1962–1963) stated that the 5mppcf standard rests on shakier evidence compared to other such recommendations [75]. 

While the OSH Act marked the beginning of unified regulation for asbestos, asbestos control would not have been such an urgent priority had it not been for the widespread notoriety asbestos received as a result of the 1965 Conference on the Biological Effects of Asbestos. This Conference organized by Professor Irving Selikoff and Jacob Churg documented the need for swift action on asbestos and a more rigorous approach to occupational safety and health [58]. Asbestos regulation in the U.S. emerged from the wilderness, and asbestos was the very first substance to be addressed as a complete standard to OSHA.

In the first 25 years of the OSH Act, asbestos control moved with great speed than at any previous time. NIOSH produced two Criteria for a Recommended Standard, one Current Intelligence Bulletin, provided four Congressional testimonies, eight scientific policy statements and over 600 published reports [76]. OSHA during this same time period issued two Emergency Temporary Standards (ETSs) for asbestos, issued three major Notices of Proposed Rulemaking (NPRMs), three final asbestos standards, and 31 Federal Register notices related to asbestos regulation [77].

The first regulation on asbestos by OSHA for general industry was issued on 29 May 1971. This asbestos standard derived from consensus standards previously adopted by the Walsh-Healy Act, which used the American Conference of Governmental Industrial Hygienists threshold limit from 1968 [78,79].

Six months after NIOSH became operational, Dr. Marcus Key, Director of NIOSH, sent a letter to OSHA warning that the initial OSHA consensus based standard for asbestos was too high and should be reduced from 12 fibers/m^3^ > 5 microns in length to 5 fibers/m^3^ [80]. OSHA had also been petitioned by the AFL-CIO to reduce worker exposure to asbestos, based on a report from Dr. Irving Selikoff and staff of the Mount Sinai School of Medicine. The AFL-CIO recommended setting a work practice standard rather than simply a standard with just a PEL [77]. 

As a result, on 7 December 1971, OSHA issued an Emergency Temporary Standard (ETS) for asbestos of 5 million fibers/m^3^ based on a count of fibers greater than 5 μm in length and with no peak exposures over 10 fibers/m^3^ for up to 15 min in an hour for up to 5 h in an 8 h day, with mandatory respirators where engineering controls were not feasible. OSHA justified this emergency standard saying: “*…working conditions constituted a grave danger and that an ETS was necessary*” [81]. The procedure of using the ETS was short lived after OSHA issued a second ETS in 1983 [82] that was immediately challenged by the asbestos industry in court and thrown out [83]. OSHA never attempted to use the ETS authority again [77].

On 25 February 1972, three months after Dr. Key’s letter to OSHA, NIOSH’s first Criteria Document for a recommended asbestos standard was transmitted to OSHA. This Criteria Document included recommendations for work practices, environmental monitoring, medical surveillance, labeling, personal protective equipment, clothing, record keeping, and recommended OSHA set a PEL of 2 fibers/m^3^ based on a count of fibers greater than 5 μm in length with peak exposures limited to not over 10 fibers/m^3^ (greater than 5 μm). [Note: PEL = Permissible Exposure Limit, a term used by OSHA and to distinguish the NIOSH recommendation from an OSHA standard in the 1980s NIOSH changed their use of PEL to recommended exposure limit (REL). The NIOSH recommendation also called for periodic medical examinations and specified respirator types required by concentrations in excess of the PEL [84]. OSHA then issued a final asbestos standard on 7 June 1972 [85]. This new standard set a PEL of 5 fibers/m^3^ based on a count of fibers greater than 5 μm in length to occur immediately, and a lower PEL of 2 fibers/m^3^ > 5 μm in length to become effective 1 July 1976.

In 1973 the Environmental Protection Agency (EPA) banned spray-applied surfacing asbestos-containing materials in fireproofing/insulation on buildings, structures, pipes, and conduits that contain material of more than 1% asbestos (40 CFR Part 61, Subpart M; 61.146) [86]. Then in 1975 the EPA banned installation of asbestos pipe and block insulation as facility components, and on boilers or hot water tanks when the material was either pre-formed (molded) and friable or wet applied such that it could become friable after drying (40 CFR Part 61, Subpart M) [86]. In 1978, the EPA expanded the ban to all spray-applied surfacing materials not covered in the 1973 spraying ban (40 CFR Part 61, Subpart M) [86].

In 1975, NIOSH convened a meeting with other government agencies, university scientists, industry representatives, and labor union officials to discuss the hazards of asbestos in brakes. It reported average peak asbestos air concentrations for specific brake servicing operations, including blow-out, grinding, and beveling of new truck brake linings within 10 feet of the operator were as high as 37.3 fibers/m^3^ (>5 μm in length). This prompted NIOSH to issue a Current Intelligence Bulletin (CIB) on asbestos in brakes giving specific recommendations for protecting workers [87]. NIOSH felt it unnecessary to initiate an epidemiologic study at this time, because it was well documented in the scientific literature that asbestos is carcinogenic and the concentrations reported in the CIB were clearly high enough to place those exposed to brake dust at risk of asbestos-related diseases, including cancers. However, the issuance of the CIB did not stop a major lobbying effort by the brake manufacturers to exonerate the dangers of asbestos in brakes that has continued through today [88]. The action of the asbestos brake manufactures as described by Rosner and Markowitz serve as an illustrative example of why asbestos regulations are so difficult to achieve and why the U.S. is one of the few developed countries in the world that has not yet banned asbestos. 

In 1975, OSHA declared asbestos a human carcinogen and published a Notice of Proposed Rule Making (NPRM) intending to revise the asbestos standard for general industry but not for construction, with a new PEL of 0.5 fibers/m^3^ [89]. This proposed rulemaking was never promulgated due to successful industry lobbying which led to four Senators and two Congressmen objecting that the proposal was economically infeasible for industry. Due to lack of political support, OSHA did not hold any hearings and thus dropped the new standard entirely [77].

In 1976, NIOSH issued a “*Revised Recommended Standard for Asbestos*” to update its 1972 recommended standard. NIOSH concluded that exposure to all types of asbestos fibers caused cancer and asbestosis and presented evidence showing that mesothelioma and lung cancers were induced following as little as 1 day of exposure to asbestos inhaled by animals. These data reaffirmed that asbestos-induced diseases were dose-response related and that excess cancers were found at all fiber concentrations, showing no evidence for a threshold or a “*safe*” level of asbestos exposure. 

These findings caused NIOSH to recommend a new REL based on the lowest level detectable by available analytical techniques. At the time, phase contrast microscopy (PCM) was found to be the only generally available and practical analytical technique. Using PCM, NIOSH recommended the concentration should be set at 0.1 fibers/m^3^ > 5 μm in length over an 8 h TWA with no greater than 0.5 fibers/m^3^ > 5 μm in length for a 15-min sampling period. NIOSH concluded this recommendation would (1) protect against the non-carcinogenic effects of asbestos; (2) materially reduce the risk of asbestos-induced cancer, with the caveat that “*only a ban can assure protections against carcinogenic effects of asbestos*”; and (3) be measured by techniques that are valid, reproducible, and available to industry and official agencies. NIOSH recognized that “*some difficulties arise in that specific work practices and innovative engineering control or process changes are needed,” but “because of the well-documented human carcinogenicity from all forms of asbestos, these difficulties should not be cited as cause for permitting continued exposure to asbestos at concentrations above 100,000 fibers > 5 μm in length/m^3^ (0.1 fibers/cc > 5 μm in length)*” [6]. This represented the first time a U.S. Government Agency had suggested a complete ban on asbestos in the workplace, a policy that NIOSH continues to advocate. 

In 1977 another partial ban on asbestos was issued by the Consumer Product Safety Commission (CPSC) when it set forth a ban on artificial fireplace embers made with asbestos and all joint wall patching compounds containing asbestos [90]. 

Joseph A. Califano Jr., Secretary of Health, Education, and Welfare, held a press conference on 26 April 1978 in Washington, DC, warning on the dangers of handling asbestos and announced that a 3-page “*physician advisory*” was to be sent to all 400,000 physicians in the U.S. from the Surgeon General of the United States regarding the hazards of asbestos. Prior to this no systematic government effort had ever been made to warn physicians about the dangers of asbestos. The advisory stated that those exposed to asbestos may be at risk of asbestosis and at risk of developing cancer, including malignancies of the lung, mesothelioma, and gastro-intestinal tract cancers. The advisory suggested that physicians do several things for those potentially exposed to asbestos, including (1) taking an occupational or exposure history; (2) carefully managing lung disease; (3) placing emphasis on smoking cessation since the combination of smoking and being exposed to asbestos greatly increased a worker’s risk of developing cancer beyond that of just smoking or asbestos exposure alone; and (4) screening for cancer. Additionally, the advisory emphasized primary prevention from asbestos exposures through ventilation and engineering controls at the worksite and the use of respirators. Secretary Califano stated the exact number of Americans exposed to asbestos was unknown, but could be as high as 8 to 11 million since the start of World War II and that health affects occurred after a long latency period of 15 to 35 years or more from initial exposure. The “*physician advisory*” stated “*exposures as short as a month may result in disease many years later*” [91].

In 1979, Dr. Eula Bingham, Director of OSHA, and Dr. Anthony Robbins, Director of NIOSH, formed a joint Working Group to evaluate the effectiveness of the OSHA Asbestos Standard. This Working Group affirmed that there was no safe level of exposure to asbestos; that the OSHA PEL was inadequate; and there is no level of exposure below which clinical effects do not occur, as evidenced by asbestos-related disease in members of asbestos-worker households and in persons living near asbestos-contaminated areas. Also, the Committee stated:

“*Due to the sampling and analytical difficulties concerning asbestos, manufactures of asbestos-containing products such as construction materials should perform detailed monitoring of exposures which could result from all foreseeable uses of their products, including misuse. This monitoring should include electron microscopy to identify fiber types mix and exposures to fibers less than 5 μm in length. This monitoring data should accompany these products downstream so the users not only know that asbestos exposures may occur, but also know the nature of potential exposures.*”

With these and other findings, the Committee recognized the need for curtailing asbestos in the workplace [92].

Despite these gains in knowledge, both the setting of effective standards to protect individuals from asbestos exposures and the banning of asbestos in the U.S. proved difficult. Professors David Rosner and Gerald Markowitz, distinguished public health historians have written that:

“*One major reason for the delay in banning this known carcinogen: private industry trade groups stifled earlier efforts at regulations.*”

[88]

A major impediment to setting asbestos standards in the United States resulted from a U.S. Supreme Court ruling in 1980 on benzene. In *Industrial Union Department v. American Petroleum Institute* the Court held:

*“Therefore, before the Secretary can promulgate* any *permanent health or safety standard, he must make a threshold finding that the place of employment is unsafe in the sense that significant risks are present and can be eliminated or lessened by a change in practices.*”

[93]

A key consequence of this decision was that all subsequent proposed standards would require a risk analysis, not only of risk at the current level of exposure but also at the level proposed for the new standard. Thus a quantification of risk must justify any new standard. 

Following this unfortunate ruling, a new NPRM on asbestos was published on 10 April 1984. Following multiple hearings, OSHA published two new asbestos standards on 20 June 1986 covering general industry (29 CFR 1910.1001) and another on construction, which reduced the PEL to 0.2 fibers/m^3^ (29 CFR 1926.1101). OSHA’s risk assessment determined the standards would prevent 7815 cancer deaths over a 45-year working lifetime, or in terms of rates OSHA estimated that the cancer risk to workers exposed to asbestos for 45 years at 0.2 f/cc is approximately three cancer deaths per 1000 exposed workers. While OSHA still determined that 0.2 fibers/m^3^ was a “significant” risk, it concluded it could not be lowered further due to technological and economic feasibility. However, the U.S. Court of Appeals for the District of Columbia issued a decision on this PEL asking OSHA to explain why 0.1 fibers/m^3^ was infeasible, because some occupations already were exposed below the 0.1 fibers/m^3^. To address the Court’s concerns, two years later OSHA added an excursion limit of 1 fibers/m^3^ for 20-min along with other ancillary provisions to protect the worker from this undue risk at the 0.2 fibers/m^3^ PEL. [77]. 

Since the changes to the 0.2 fibers/m^3^ PEL did not entirely address the Courts question, OSHA continued to explore ways to protect workers from asbestos. On 20 July 1990, OSHA proposed a new PEL be adopted of 0.1 fibers/m^3^ as had originally been recommended by NIOSH in 1976; adding specific work practices for most asbestos abatement maintenance and repair activities regardless of exposure levels; requiring a designated “competent person” for all abatement; hold building owners accountable for communicating asbestos hazards in their buildings; and for OSHA to maintain the regulatory scheme on the type of work performed, rather than allow the industry sector to determine which standard (general industry or construction) applied [77]. Thus, a final standard was published for general industry and a separate one for construction [94]. Not only did these standards cut the PEL in half, they required all employers communicate asbestos hazard information to each other as well as to their employees. In construction projects the general contractor was required to exercise general supervisory authority under the standard as well as to maintain compliance and could require all asbestos contractors to maintain compliance. The owners of buildings and facilities who were employers were held responsible to identify asbestos and warn contractors and employees. Although the standard did not require owners to test for asbestos, it did require them to assume all thermal system insulation, sprayed on or troweled onto surface material and flooring material installed prior to 1981 may contain asbestos unless testing proved otherwise [94].

In 1989, the EPA took one of the most dynamic steps to ban asbestos in the U.S., issuing a final rule banning most asbestos-containing products under Section 6 of the Toxic Substances Control Act (TSCA). This was a staged ban aimed at banning future manufacturing, importing, processing, and distributing [95]. However, this ban was short-lived. Industry appealed the ban in the courts, and on 18 October 1991, the rule was vacated and major parts of it remanded by the United States Court of Appeals for the Fifth Circuit. Thus, due to the Fifth Circuit’s decision and subsequent clarification most existing asbestos-containing products were not banned. Some examples of asbestos containing products that were allowed to remain in U.S. markets as a result of this decision include:

○ Cement corrugated sheet
○ Cement flat sheet
○ Clothing
○ Pipeline wrap
○ Roofing felts
○ Vinyl floor tile
○ Cement shingle
○ Millboard
○ Cement pipe
○ Automatic transmission components
○ Clutch facings
○ Friction materials
○ Disk brake pads
○ Drum brake linings
○ Brake blocks
○ Gaskets
○ Non-roofing coatings
○ Roof coatings

The asbestos-containing products banned by EPA under the Toxic Substances Control Act (TSCA) were limited to a mere handful of product types, including: corrugated paper; rollboard; commercial paper; specialty paper; and flooring felt. In addition, the regulation continued to ban the use of asbestos in products that have not historically contained asbestos, otherwise referred to as “*new uses*” of asbestos. Also, under the Clean Air Act (CAA) other asbestos-containing uses were banned including: asbestos pipe insulation; asbestos block insulation on facility components, such as boilers and hot water tanks; spray-applied surfacing asbestos-containing materials; and spray-on application of materials containing more than 1% asbestos to buildings, structures, pipes, and conduits unless meeting certain conditions specified under 40 CFR 61, Subpart M [95].

OSHA asbestos regulations broke ground in many ways by being heavily oriented toward engineering and work practices controls, although they were very slow in development and this delay contributed to continuing exposure to workers [77]. As Martonik et al. [77] stated:

“*It is unlikely that the authors of the OSHA Act envisioned an agency working on asbestos standards from its inception in 1971 to 1998.*”

## 6. Discussion

Scientific and regulatory agencies internationally and in countries around world, including agencies of the United States government—NIOSH, OSHA, and EPA—have determined that asbestos is a proven human carcinogen, that no form of asbestos is safe, that no level of exposure to asbestos is safe, and that there is no way to work safely with asbestos. In consequence of these recognitions, approximately 55 countries around the have banned asbestos. The Collegium Ramazzini has also weighed in and in 1999, made its first of its many calls for a universal ban on asbestos mining, manufacture, and all uses of asbestos [96]. The Collegium Ramazzini stated;

“*The profound tragedy of the asbestos pandemic is that all illnesses and deaths related to asbestos are preventable.*”

The Collegium Ramazzini added that even if all asbestos use were to cease today there would be no noticeable decrease in diseases or deaths for at least 20 years [97]. Asbestos diseases are still with us and continue to increase. 

The Institute for Health Metrics and Evaluation, supported by the Gates Foundation, estimates that approximately 194,000 asbestos-induced cancer deaths occurred globally in 2013, up from around 94,000 in 1990, an increase of 109.6% [98]. Considering only mesothelioma, a signal tumor for asbestos [99] the number of deaths in the US have continued to increase in the last 16 years from 2479 to 2597. It was further noted that the trend of “*continuing occurrence of malignant mesothelioma deaths in persons aged < 55 suggest ongoing inhalation exposures to asbestos fibers*” [3]. Since mesothelioma represents a marker tumor [99] for which it is estimated that for every mesothelioma there are six asbestos-induced lung cancers, their deaths only represent the “tip-of-the-iceberg” for asbestos-induced cancers [100].

The failure of the United States to ban asbestos reflects the enormous power of the asbestos industry and its political allies to act against the best interests of public health and to place short-term profits ahead of human well-being. The primary strategy used by the asbestos industry in this struggle has to manufacture doubt – to create false controversies around questions that were settled long ago by reputable and independent scientists. These tactics are similar to those used by the tobacco industry and other polluting industries. So long as the courts in the United States allow corporations and individuals to make large, unrestricted donations to political campaigns, the potential for this sort of corruption will continue.

The road to banning asbestos in the U.S. has a new glimmer of hope under the 2016 revision of the Toxic Substance Control Act (TSC Act), the Senator Frank R. Lautenberg Chemical safety for the 21st Century Act (known as the Frank R. Lautenberg Chemical Safety for the 21st Century Act):

“*Under the new Act, a risk evaluation of asbestos is to be completed within three years and, if asbestos is found to be an ‘unreasonable risk to humans and the environment,’ the EPA is required to mitigate that risk, possibly through a ban, within two more years.*”

[88]

Responsibility for enforcement of this new law now rests in the hands of the Trump Administration. The tragedy of disease caused by asbestos is that it is entirely preventable. The cause is known, and a complete ban on all manufacture, import and use of asbestos is a proven effective preventive strategy. There has been sufficient science available for many decades to guide the prevention of all asbestos-related diseases. The failure to ban asbestos in the United States is a national scandal and an affront to morality and human decency.

## 7. Conclusions

Asbestos is a proven human carcinogen. All forms of asbestos can cause cancer. No level of exposure to asbestos is safe. A complete ban on all manufacture and use of asbestos worldwide is the only strategy that will effectively end the global pandemic of asbestos-related disease.

The United States has tried for decades to impose a ban on all manufacture, import and use of asbestos, but these efforts have failed through a combination of OSHA’s ineptness, industry’s lobbying power, and the Fifth Circuits Court’s 1991 decision not to uphold the ban on asbestos that was proposed by the Environmental Protection Agency. The consequence of these delays in banning asbestos in the United States is that thousands of workers have needlessly died from asbestos-related disease and tens of thousands more have suffered disease and disability. It is to be hoped that asbestos will finally be banned in the United States under the Senator Frank R. Lautenberg Chemical safety for the 21st Century Act.
